# Textured Stainless Steel as a Platform for Black Mg_2_Si/Si Heterojunction Solar Cells with Advanced Photovoltaic Performance

**DOI:** 10.3390/ma15196637

**Published:** 2022-09-24

**Authors:** Alexander V. Shevlyagin, Vladimir M. Il’yaschenko, Aleksandr A. Kuchmizhak, Eugeny V. Mitsai, Andrey V. Amosov, Semyon A. Balagan, Sergei A. Kulinich

**Affiliations:** 1 Institute of Automation and Control Processes, Far Eastern Branch, Russian Academy of Science, 5 Radio Str., 690041 Vladivostok, Russia; 2 Pacific Quantum Center, Far Eastern Federal University, 690041 Vladivostok, Russia; 3 Research Institute of Science & Technology, Tokai University, Hiratsuka 259-1292, Kanagawa, Japan

**Keywords:** antireflection, silicide, solar cell, stainless steel, surface texturing

## Abstract

This paper reports on a facile bottom-up method for the direct integration of a silicon (Si)-magnesium silicide (Mg_2_Si) heterojunction solar cell (HSC) with a textured rear reflector made of stainless steel (SS). Modified wet chemical etching and post processing of SS substrates resulted in the formation of both a rough surface texture and diffusion barrier layer, consisting of magnetite (Fe_3_O_4_) with reduced optical reflection. Then, Si, Mg_2_Si and CaSi_2_ layers were stepwise thermally evaporated onto the textured SS surface. No traces of Fe and Cr silicide phases were detected by Raman spectroscopy, confirming effective suppression of impurity diffusion from the SS to the upper layers at least at temperatures required for Si deposition, as well as Mg_2_Si and CaSi_2_ formation. The obtained black-SS/Fe_3_O_4_/Si/Mg_2_Si/CaSi_2_ sample preserved, to some extent, its underlying textured morphology and demonstrated an averaged reflection of 15% over the spectral range of 200–1800 nm, while its prototype HSC possessed a wideband photoresponse with a photoelectric conversion efficiency of 7.5% under AM1.5 illumination. Moreover, Si layers deposited alone onto a black-SS substrate demonstrated competitive antireflection properties compared with black Si (b-Si) obtained by traditional top-down etching approaches, and hybrid b-Si/textured-SS structures with a glue-bonded interlayer.

## 1. Introduction

Building-integrated photovoltaics (PV) is an essential part of modern solar power technology moving towards net-zero-energy and zero-carbon-emission engineering [1,2,3,4,5,6,7,8]. The utilization of conventional construction materials to substitute crystalline silicon, glass or solar-grade stainless steel (SS) substrates seems to be the most cost-effective solution [9,10,11] for large-scale integration of solar cells (SCs). 

Currently, SS is used as a part of complex layered substrates for amorphous and crystalline SCs because of its merits, such as good flexibility, high thermal and chemical stability, low-cost and good electric conductivity. To adopt rolled (or high-gloss) SS substrates for PV demands, the steel industry offers the deposition of intermediate layers for simultaneous surface smoothing, back electrode insulating, and preventing diffusion of various impurities from the SS substrate into the light absorbing layer of thin-film solar cells (TFSCs) for each semiconducting material [12,13,14]. Other applications of SS in the PV industry, except for ancillary components, frames, fasteners and connectors, include substrates for solar spectral selective absorbing coatings used in thermo-solar systems [15,16,17]. In all of the above-mentioned cases, SS is used as a supporting substrate for flexible solar cell production or deposition of metallic back optical reflectors [18,19,20]. The use of SS substrates instead of common crystalline, glass or polymer substrates typically leads to reduced photoelectric conversion efficiency (PCE) of the resulting device, regardless of the particular solar light absorbing semiconducting materials used. This is explained by the relatively lower crystallinity, contaminant diffusion and high surface roughness of devices prepared on SS platforms.

Nevertheless, the feasibility of using an SS substrate was demonstrated for Si, amorphous Si (a-Si), copper indium gallium selenide (CIGS), and perovskite solar cells [21,22,23,24]. However, such solutions suffer from either the formation of iron- and chrome-related deep-level “killers” or metal silicide precipitation, which accompanies growth of the absorbing layers atop SS due to the intense diffusion of contaminants from SS. In addition, PCE values of the produced SCs are highly sensitive to surface roughness. As a result, various diffusion barriers and planarization layers are necessary to preserve competitive power efficiency at the cost of additional deposition steps. 

Another important aspect limiting PCE is associated with TFSC design itself. The thinner the solar light absorbing layer, the more complex the light management strategy that has to be applied, to compensate for the decrease in the generated photocurrent. Texturing of the Si surface is an efficient approach that permits the reduction of the thickness of the light absorbing layer while retaining the same level of light absorption and photocurrent generation [25,26]. Thus, integration of an SS substrate with so-called black materials (e.g., black Si, Si pyramids and vertical nanowire arrays) appears to be a natural solution to achieve high PCE using low-cost supporting substrates. In this light, a very smart but unpractical example of such an integration was demonstrated by Omar et al. [27,28] who independently produced a textured rear reflector and b-Si by wet chemistry etching of SS and Si substrates followed by glue bonding. Actually, an increase in light absorption was obtained compared with freestanding b-Si; however, there were no remarks regarding the PCE of the proposed SS/b-Si device. In addition, one can assume that glue bonding is hardly compatible with both reliable back electrode fabrication and outdoor SC operation, due to inevitable heating under direct sunlight.

In this work, we implemented the “bottom-up” approach to directly integrate Si layers with a textured rare reflector made of an SS substrate that also served as a back electrode. Moreover, the proposed advanced two-step wet-etching protocol resulted in covering the textured SS surface with Fe oxide (Fe_3_O_4_), which acted as an effective diffusion barrier during Si overgrowth. The resultant SS/Fe_3_O_4_/Si templates demonstrated competitive antireflection properties without needing to deposit any intermediate layers. In addition, we further exploited our concepts of the black silicide [29], which deals with semiconducting magnesium silicide (Mg_2_Si) formation atop a textured Si surface, and optically transparent calcium disilicide (CaSi_2_) [30] that acts as a top electrode, to demonstrate a prototype Si-silicide heterojunction solar cell (HSC) with the black-SS/Fe_3_O_4_/Si/Mg_2_Si/CaSi_2_ structure. The former concept is devoted to the silicidation of the Si surface textures, which results in the preservation of the original morphology, while providing unprecedented near-infrared (NIR) antireflection performance. The latter concept utilizes CaSi_2_ as Si- and silicide-compatible transparent conducting material, despite being a semimetal in nature. The designed and tested HSC demonstrated a relatively low averaged reflection (AVR) of 15% in the spectral range of 200–1800 nm with a room temperature PCE value of 7.5% registered under illumination of AM1.5. The obtained result gave a 30-fold increase in PCE compared with previously reported Si/Mg_2_Si HSC with flat geometry [31], which approaches the performance of the best ever reported Si-silicide HSC [32]. Moreover, the applicability of the black-SS/Si templates could be further extended by SS foil texturing, which opens opportunities for flexible optoelectronic devices. 

## 2. Materials and Methods

**Figure 1:**
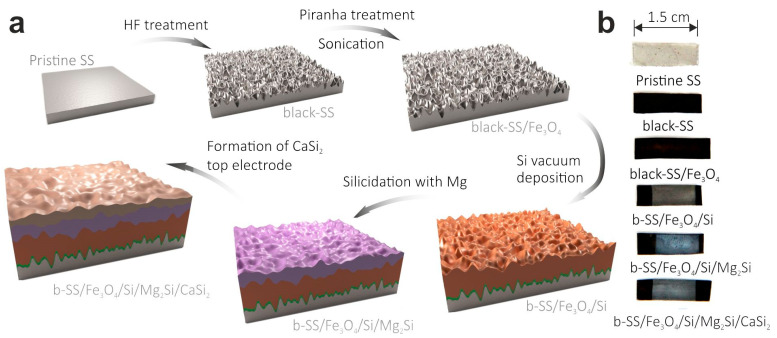
(**a**) Illustration of step-by-step fabrication of the black-Mg_2_Si/Si heterostructure with a top semimetal CaSi_2_ transparent electrode grown on textured stainless steel substrate. (**b**) Photographs of corresponding SS samples at each fabrication step.

All experiments on the fabrication of textured rear back electrodes were carried out with high-gloss SS304 (NLMK, Lipetsk, Russia) specimens (referred to as pristine SS in Figure 1a,b) with 15 × 5 × 0.5 mm^3^ (L × W × d) dimensions. At the first stage, SS304 samples were immersed in deionized water (DIW, ChemElectronics, St. Petersburg, Russia) diluted HF (LenReactives, St. Petersburg, Russia) solution for 30 min (resulted samples are referred to as black SS or b-SS in Figure 1a,b) followed by treatment in an HNO_3_ (LenReactives, St. Petersburg, Russia) water solution for surface passivation in accordance with the well-known protocol of superhydrophobic SS surface preparation [33]. Due to the lack of information regarding antireflection properties of textured SS samples, which were mainly produced by laser-assisted methods [34], additional experiments were performed after etching with HF to achieve the best optical performance. Sonication in Piranha solution (H_2_O_2_:H_2_SO_4_ ratio of 1:1, LenReactives, St. Petersburg, Russia) was applied to remove reaction products after texturing with HF, instead of treatment with HNO_3_ used for the b-SS samples. The resultant structures are referred to as b-SS/Fe_3_O_4_ in Figure 1a,b. In addition, an SS sample treated solely with HF was prepared for comparison. 

Silicon layers were deposited on the treated SS substrates in a vacuum chamber at a base pressure of 10^−6^ Torr. The chamber was equipped with a DC-heated Si evaporation source, quartz microbalance sensor, and rotating samples holder with resistive heating option up to 900 °C. The Si deposition rate was calibrated to be ~6 nm/min, while the thickness of layers was varied from 50 to 1000 nm. 

Surface morphology and hemispheric total reflectance in the spectral range of 200–1800 nm were chosen as markers towards maximizing antireflection performance of both bare and Si-covered textured SS samples. The resulting texture morphology was investigated by atomic force (AFM, Solver P47, NT-MDT, Moscow, Russia) and scanning electron (SEM, Hitachi S3400, Tokyo, Japan) microscopy, while optical properties were measured on a spectrophotometer with an integrating sphere (Cary 5000, Varian, Palo Alto, CA, USA) under normal incident conditions. While surface roughness (RMS) was directly available from AFM data, several key parameters were missing, which could have helped to achieve a trade-off between SC short-circuit current and open-circuit voltage that depends on the texture’s morphology, rather than simply on its RMS value [35]. Thus, for correlating morphology and optical properties, we used texture angle calculations derived from surface topography images, with calculation details being available elsewhere [36].

Solid phase epitaxy technique was used to produce both solar light absorbing and top electrode layers deposited in vacuum. First, a 100-nm-thick magnesium (Mg) film was deposited at room temperature with a 25 nm/min evaporation rate from K-cell, followed by annealing at 350 °C for 5 min. This procedure has previously been shown to result in silicidation of Si surface texture and black Mg_2_Si formation [35]; this step is shown in Figure 1a, while the optical photograph of the produced sample marked as b-SS/Fe_3_O_4_/Si/Mg_2_Si is shown in Figure 1b. At the final fabrication stage (see Figure 1a), a bilayer of Si and Ca (K-cell, 25 nm/min) was deposited atop the Mg_2_Si light absorbing layer at room temperature followed by rapid thermal annealing at 600 °C for CaSi_2_ formation [36]. Thus, the final structure of the black-Mg_2_Si/Si heterostructure with transparent top and textured back electrodes made of CaSi_2_ film and SS304 substrate, respectively, is snapshotted in Figure 1b, and further referred to as b-SS/Fe_3_O_4_/Si/Mg_2_Si/CaSi_2_. To check whether CaSi_2_ acts as an effective transparent electrode, uncovered b-SS/Fe_3_O_4_/Si/Mg_2_Si samples were grown for comparison. Raman spectra (473 and 633 nm CW laser pump, NTEGRA Spectra II, NT-MDT, Zelenograd, Russia) were recorded for all samples to confirm phase composition and layered structure. 

Ultrasonic welding with Al wires was used for the resulted SC prototype packing before PV characterization. In the case of samples uncovered with CaSi_2_, Al ring electrodes were formed by magnetron sputtering. Current density–voltage (J-V) characteristics were obtained with a DC current source meter (Keithley Instruments, Cleveland, OH, USA) in the dark. Spectral response was evaluated using a calibrated Xe lamp (Hamamatsu, Tokyo, Japan) optically connected to a monochromator (Solar Tii, MS3504i, Minsk, Belarus) equipped with an optical filter bank (Semrock Inc., New York, NY, USA), synchronous detection system with lock-in amplifier (SRS830, Stanford Research System, Sunnyvale, CA, USA) and an optical chopper for light modulation (Thorlabs, Newton, NJ, USA). Current density vs. voltage (J-V) curves were measured under AM1.5G illumination for calculating the PCE of the obtained SC prototypes.

## 3. Results and Discussion

**Figure 2:**
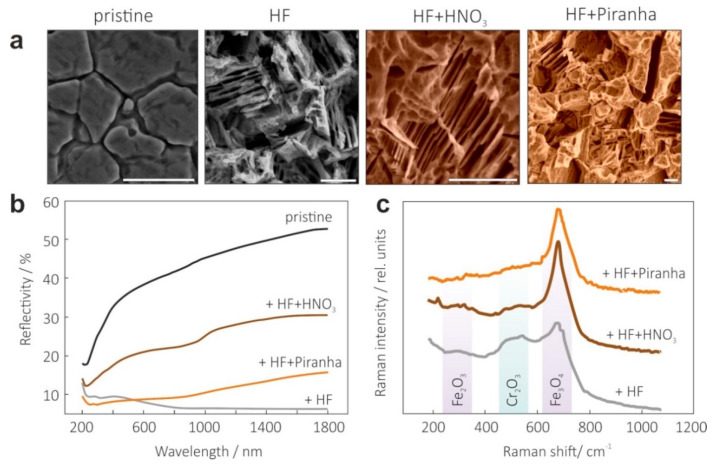
Summary of acid-induced texturing and stabilization on SS surface. (**a**) Evolution of surface morphology after different treatments shown in a series of top-view SEM images. Scale bar indicates 10 µm. (**b**) Optical reflection spectra of pristine SS and those treated under different conditions. (**c**) Probing the changes in surface composition of the SS samples treated under different acid solutions with Raman spectroscopy at 473 nm pump.

**Figure 3:**
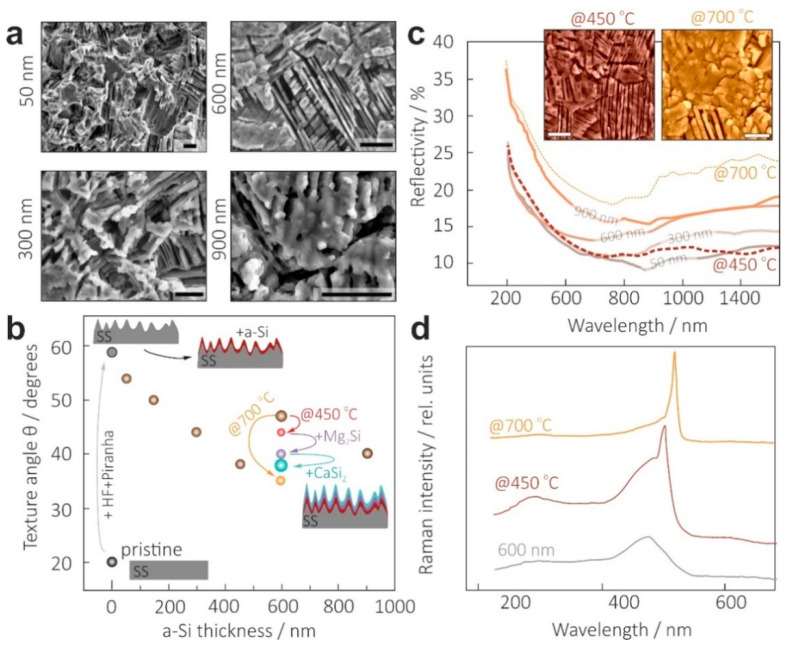
Optimization of the growth temperature and thickness of the Si layer capping b-SS. (**a**) Top-view SEM images showing morphology variation and surface flattening of the b-SS samples with an increase in Si layer thickness from 50 to 900 nm. Scale bar indicates 10 µm. (**b**) Texture angle evolving upon increase in Si thickness (brown markers) as well as after each fabrication step of the prototype SC device (colored markers). (**c**) Influence of Si layer thickness and growth temperature on the optical properties of the b-SS/Si template structure. The insets show surface morphology of the b-SS/Si sample heated at 450 and 700 °C. Scale bar indicates 2 µm. (**d**) Crystallinity of Si layers deposited under different growth conditions accessed by Raman spectroscopy.

The surface of the HF-etched SS sample (frame two in Figure 2a) demonstrated a relatively bare structure at the microscale presented by so-called “gullies” and “plateaus” typical of an acid-etched metal surface [37]. The sample possessed the highest surface roughness characterized by a very high texture angle (see Figure 3b) and the lowest AVR of 7.2% in the spectral range of 200–1800 nm, in accordance with Figure 2b. However, brief examination showed that this optical performance was obtained at the cost of black powder formation, which covered the microstructure of the SS substrate after HF treatment. This powder is mechanically unstable and can be easily removed with scotch tape. Raman measurements, shown in Figure 2c, revealed that a black powder and the SS surface microstructure mainly consisted of Fe (Fe_2_O_3_, Fe_3_O_4_) and Cr (Cr_2_O_3_) oxides with corresponding Raman bands centered at 291, 347, 551 and 679 cm^−1^ [38]. It is known that SiO_2_-based additives [37] or an additional passivation step [39] can result in more uniformly textured morphology formation due to promoted corrosion in H_2_SiF_6_ acid compared with HF, or surface stabilization after HNO_3_ treatment. Indeed, Figure 2c clearly shows that the HNO_3_ treated SS304 sample demonstrated lower Fe_2_O_3_ and Cr_2_O_3_ related Raman features with a pronounced Fe_3_O_4_ contribution. Importantly, no black powder was observed, suggesting surface conditions to be acceptable for further vacuum deposition of other layers. Unfortunately, optical examination, presented in Figure 2b, revealed significant degradation of antireflection performance, with an AVR of 24.4% regardless of HNO_3_ concentration and passivation time. Treatment in Piranha solution is known to be efficient for surface passivation and removal of various contaminates including metallic ones. In accordance with SEM (right-most frame of Figure 2a) and Raman studies (Figure 2c), sonication of the b-SS samples in Piranha solution resulted in both hierarchical micro- and nano-texturing of the surface and its stabilization with Fe_3_O_4_, seen in Raman spectra, without any evidence of contribution from Fe_2_O_3_ and Cr_2_O_3_. Moreover, after optimization of sonication time and concentration of Piranha solution, this procedure was found to provide an AVR of 11%, that was slightly worse than compared with the value achieved for HF-etched SS samples (see Figure 2b). Additionally, Fe_3_O_4_ covering could be applied to suppress impurity diffusion from SS304 [13,40] into the upper Si and silicide light-absorbing layers, as will be demonstrated below.

Next, we focused on vacuum deposition of Si layers atop a textured back electrode made of SS304 samples with a b-SS/Fe_3_O_4_ structure. In doing so, several key points affecting optoelectronic properties of the proposed SC material design had to be addressed. First, combined optical reflectance and electrical conductivity measurements were performed to determine an optimal thickness of Si layer, which could be characterized by both preservation of the b-SS/Fe3O_4_ antireflection and electrical isolation of the upper Mg silicide layer. The latter is a crucial issue resulting from both high surface roughness of the b-SS/Fe_3_O_4_ and high conductivity of the Fe_3_O_4_ layer [41]. Secondly, Raman spectroscopy was used for monitoring possible Fe and Cr diffusion, which could result in silicidation and silicide precipitation in the Si grown layers (thus, strongly affecting the dark current of any Si SC supported by SS substrate). In addition, regarding further silicidation of b-SS/Fe_3_O_4_/Si structures with Mg, recent theoretical calculations predicted that Fe- or Cr-doped Mg_2_Si demonstrates semimetallic or metallic behavior, respectively [42], thus, deteriorating its light absorbance due to the absence of a band gap. Optical reflectance data (Figure 3c) showed that regardless of its thickness, Si deposition onto b-SS/Fe_3_O_4_ inevitably deteriorated antireflection properties. More importantly, at least a 350-nm-thick Si layer is required for solid isolation of the grown film from b-SS/Fe_3_O_4_ substrate. The morphological evolution of the b-SS/Fe_3_O_4_/Si structure with the thickness of the Si layer (Figure 3a) clearly supported this point. In particular, thin Si layers do not allow the complete covering of the surface “valleys” and “plateaus”, which can cause electrical shorting. On the other hand, the thicker the Si layer, the higher the AVR observed. It is associated with smoothing of the b-SS/Fe_3_O_4_ surface, which was reflected in a pronounced decrease in texture angle, calculated from the corresponding AFM data presented in Figure 3b (brown markers). However, AVR non-monotonically depends on Si thickness with a local minimum at 600 nm (14.5%), which also correlated well with the behavior of the texture angle. SEM studies (Figure 3a) revealed that Si coverage was characterized by the formation of additional micro relief atop a relatively smooth b-SS/Fe_3_O_4_ filled with Si. A further increase in the deposited Si thickness resulted in lower texture angles (Figure 3b) and lower antireflection performance, with an AVR of 18.5% for the 900-nm-thick Si layer (Figure 3c). Thus, an 600-nm-thick Si overlayer provided a compromise between reduced optical reflection and electrical isolation for vacuum growth of the upper light absorbing and top electrode silicide layers.

To clarify the role of Fe_3_O_4_ in suppressing interdiffusion of Si, Cr and Fe atoms, Raman spectra were recorded for room-temperature deposited and high-temperature grown (up to 700 °C) Si layers on b-SS/Fe_3_O_4_ templates. No Fe and Cr silicide-related phonon lines [43,44,45] were observed for all tested samples, as can be seen in Figure 3d. Moreover, these measurements permitted optimization of the growth temperature for the Si cover layers. For instance, the higher the growth temperature, the fewer a-Si and polycrystalline fractions in deposited Si layers were observed. Despite the demonstrated ability of the Fe_3_O_4_ layer to suppress SS contaminant diffusion even at the highest growth temperature of the Si layer and its high crystallinity, the application of such a high temperature resulted in surface flattening (see insets in Figure 3c), with a higher AVR of 22.6% and optical features close to that of crystalline Si (Figure 3c). Therefore, an optimal growth temperature of 450 °C was chosen for Si growth, which provided an acceptable antireflection. Of note, the produced b-SS/Fe_3_O_4_/Si templates reached an antireflection performance comparable with that achieved for previously reported b-Si substrates glue-bonded with textured SS [27,28], yet at much lower Si consumption. 

After determining the most optimal texturing, passivation and Si overgrowth conditions to obtain Si-compatible templates for HSC development, we focused on the formation of the Mg_2_Si-based solar light absorbing layer. On one hand, Mg_2_Si possesses outstanding optoelectronic properties to be applied as a VIS-NIR light absorber and photodetector with high Si-compatibility [46,47,48,49,50,51]. On the other hand, we have already demonstrated that silicidation of the textured Si surface such as b-Si with magnesium resulted in a new wide band optical absorber called “black silicide” [29], demonstrating a very high PV potential. Thus, it seemed natural to extend this paradigm beyond b-Si substrates towards more cost-efficient ones, such as b-SS/Fe_3_O_4_/Si, considered in the present study.

**Figure 4:**
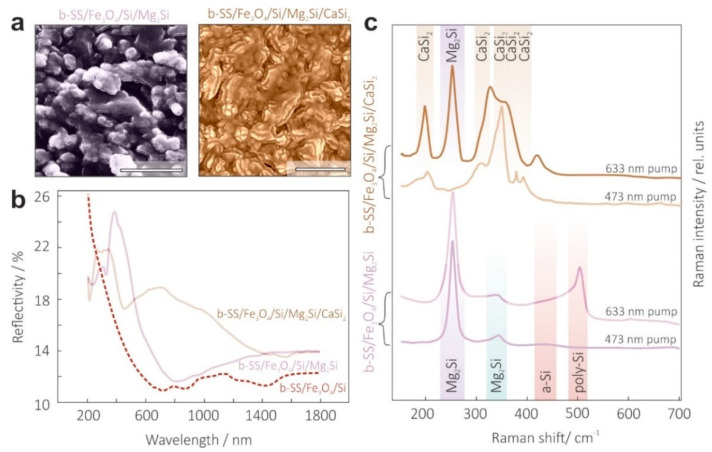
Characterization of the solar light absorbing Mg_2_Si and transparent conducting CaSi_2_ layers. (**a**) Top-view SEM images demonstrating drastically changed surface morphology of b-SS/Si templates after consequent vacuum silicidation with magnesium (**left**) and calcium (**right**). (**b**) Optical reflectance spectra of intermediate b-SS/Fe_3_O_4_/Si and b-SS/Fe_3_O_4_/Si/Mg_2_Si samples, as well as resultant b-SS/Fe_3_O_4_/Si/Mg_2_Si/CaSi_2_ SC. (**c**) Raman spectra of intermediate b-SS/Fe_3_O_4_/Si/Mg_2_Si (**bottom graphs**) and resultant SC structures (**upper graphs**) measured at 473 and 633 nm pump.

The top-view SEM image of sample b-SS/Fe_3_O_4_/Si/Mg_2_Si, shown in Figure 4a (the left frame), revealed a drastic change in surface morphology had taken place after silicidation. Raman spectra (bottom part in Figure 4c) obtained under pumping at different wavelengths, allowed us to separately probe the Mg_2_Si layer (473 nm) and underlying Si layer (633 nm) by tuning the wavelength-dependent penetration depth of the laser radiation inside the probed material. In addition, the position and FWHM of silicide-related Raman bands at 254 and 345 cm^−1^ suggested high crystal quality of the grown layers of the sample b-SS/Fe_3_O_4_/Si/Mg_2_Si. Importantly, there was still no contribution from Cr and Fe silicides, which confirmed the good potential of the Fe_3_O_4_ layer to suppress diffusion. Considering changes in the optical properties of b-SS/Fe_3_O_4_/Si templates after silicidation, outlined in Figure 4b, it can be noted that there was a slight increase in the AVR value (16%) compared with bare b-SS/Fe_3_O_4_/Si. A closer look at the morphology of the Mg_2_Si overlayer (left frame in Figure 4a) showed formation of the microflakes atop, that caused the higher reflection in the visible spectral range. 

To explore, in depth, the Si-compatible technology paradigm, we implemented the recently described concept of using a semimetal calcium disilicide top electrode [30]. Briefly, CaSi_2_ demonstrates a high figure of merit as a transparent conducting material, while its constituents are non-toxic and earth abundant. In this work, we modernized the deposition of CaSi_2_ in vacuum since it requires much higher formation temperatures in comparison with Mg_2_Si. As a result, CaSi_2_ was grown within three steps. First, a thin protective a-Si layer was deposited onto the Mg_2_Si at room temperature, followed by a thin Ca film deposition in a stoichiometric ratio, to form CaSi_2_ at the final stage upon annealing at 600 °C.

To confirm both the persistence of the Mg_2_Si layer and successful formation of a transparent CaSi_2_ electrode layer atop, Raman spectroscopy was applied at different pump wavelengths. The results are summarized in the upper graphs of Figure 4c, revealing only CaSi_2_ related bands (197, 310, 350, 378 and 390 cm^−1^ [52]) at 473 nm pump, as well as clear signatures of Mg_2_Si and Si beneath CaSi_2_ at 633 nm pump wavelength with larger penetration depth. At least five well-resolved and spectrally narrow CaSi_2_ phonon bands suggested a high-quality formation of the top electrode. 

Next, we considered the morphology and optical properties of the obtained sample b-SS/Fe_3_O_4_/Si/Mg_2_Si/CaSi_2_. Its surface structure looked walnut-like, with rather filled original b-SS “gullies”, as clearly seen in Figure 4a (the right frame). This explained the increase in total reflection, presented in Figure 4b. We speculated that the higher VIS reflection may have resulted from the transparency window of the CaSi_2_ lying in the spectral range of 550–2000 nm [30]. Nonetheless, the CaSi_2_ cover successfully fulfilled the requirements of both low sheet resistance (~35 Ω/sq.) and preservation of antireflection performance. 

**Figure 5:**
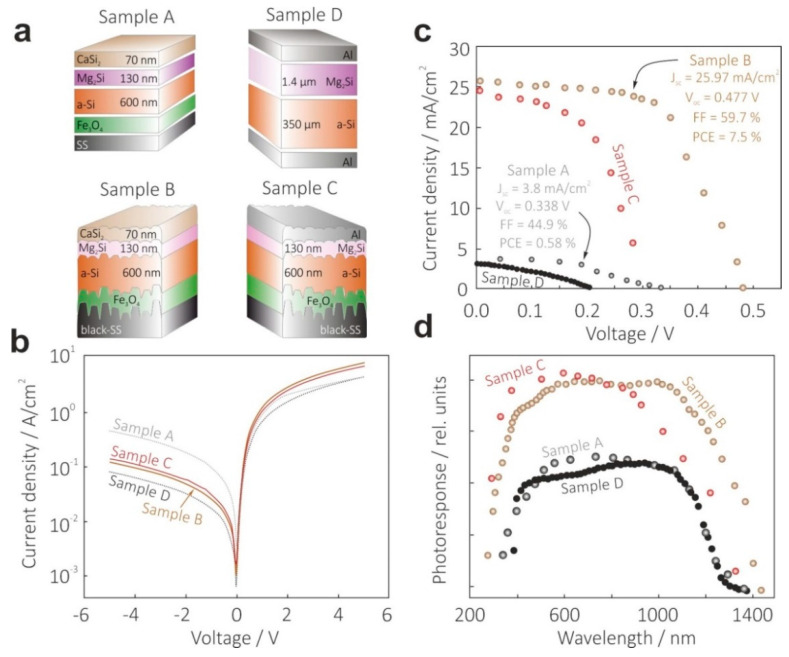
Photovoltaic performance of prototype Mg_2_Si/Si SC devices with flat and textured design. (**a**) Schematic presentation of solar cell devices with specified layered structure and thickness. (**b**,**c**) J-V curves measured under dark and AM1.5 conditions, respectively. (**d**) Room-temperature photoresponse spectra under zero-bias conditions.

Finally, to demonstrate all advantages of the proposed HSC design and systematically illustrate the influence of the textured back electrode made of SS304 substrate, black Mg_2_Si solar light absorbing layer and CaSi_2_ semimetal transparent top electrode, several device prototypes were prepared for photovoltaic examinations: flat SS/Si/Mg_2_Si/CaSi_2_ (Sample A), b-SS/Fe_3_O_4_/Si/Mg_2_Si/CaSi_2_ (Sample B) and b-SS/Fe_3_O_4_/Si/Mg_2_Si/Al (Sample C). In addition, for comparison, we also referred to our planar Al/Si/Mg_2_Si/Al, a pioneering Mg_2_Si-based HSC previously reported elsewhere [31] (Sample D). The schematic diagrams of all the above-mentioned heterojunction SC devices are illustrated in Figure 5a.

Figure 5b presents room-temperature dark J-V curves for all the devices. The most obvious striking feature was an increase in reverse saturation current for all SS304-based SC prototypes, if compared with their counterparts fabricated on Si substrate (1.9 × 10^−4^ A/cm^2^). However, Sample B demonstrated the minimal parasitic current (4 × 10^−4^ A/cm^2^) which could be directly associated with the presence of Fe_3_O_4_ diffusion barrier layer. In addition, this conclusion was supported by the observed decrease in the shunt resistance of Sample A (12 kΩ) when compared with Sample B (27 kΩ). It could be concluded that the absence of any barrier layer resulted in the formation of alternating channels for current flows due to Fe and Cr silicide precipitation during vacuum growth. The influence of the CaSi_2_ top electrode layer became clear from the series resistance behavior. There was a significant reduction in the series resistance when shifting from Al metallization with a calculated value of 27 Ω (Sample C), towards CaSi_2_ top electrode formation (Sample B) with a calculated value of 3 Ω, which resulted from a lower contact resistance in the latter case. Nevertheless, all the tested devices demonstrated clear diode characteristics with a good rectification. 

In view of the observed dark J-V characteristics, it was not surprising that Sample B showed the best PV performance under AM1.5 illumination (see Figure 5c). Indeed, the textured back electrode made of SS304 resulted in a huge increase in photogenerated current density regardless of the applied top electrode material (24.7 mA/cm^2^ for Sample C with its Al electrode, and 25.97 mA/cm^2^ for Sample B with its CaSi_2_ electrode) compared with flat configurations on both Si (Sample D, 3.3 mA/cm^2^) and SS substrates (Sample A, 3.8 mA/cm^2^). For a deeper insight into this phenomenon, we addressed the spectral photoresponse examination, presented in Figure 5d. One can see that surface texturing and subsequent black Mg_2_Si formation extended the operation spectral region to at least 1300 nm, while flat design was characterized by a small shoulder in the NIR region below the Si band gap with insignificant contribution to the overall photoresponse. Secondly, there was a difference in both photosensitivity and spectrum shape between Samples B and C, with CaSi_2_ and Al top electrodes, respectively. Aluminum contact provided higher UV-VIS photoresponse resulting from a higher transparency of Al, compared with CaSi_2_ in this spectral region. However, it was overcompensated by a much higher NIR transmittance of semimetal in nature CaSi_2_, when compared with pure metals, including Al. 

As a result, the b-SS/Fe_3_O_4_/Si/Mg_2_Si/CaSi_2_ prototype HSC was found to possess the highest PV performance with a PCE of 7.5%, J_sc_ = 25.97 mA/cm^2^, FF = 55.9% and V_oc_ = 0.477 V, thus, confirming the feasibility of the proposed approach of the direct integration of black solar light absorbers with textured back reflectors without any isolation layers for preventing uncontrolled diffusion and other accompanying issues. The PCE achieved for the black Si/Mg_2_Si tandem not only demonstrates a 30-fold increase in PV performance compared with its flat counterpart [29], but also keeps pace with other state-of-the-art SCs fabricated on other construction-compatible substrate materials. Moreover, the b-SS/Fe_3_O_4_/Si templates that were also used in the current study create an opportunity for a simple, low-cost approach for the fabrication of substrate-supported black Si structures for flexible and other feasible applications.

## 4. Conclusions

In the present work, we developed a low-cost protocol that directly integrates a stainless steel based textured back electrode to light-trapping Si surface textures. More importantly, the resulted samples with Si covers can be used as a template for Si tandem SC fabrication. To prove the concept, we created a Si/Mg_2_Si heterojunction on its surface by simple vacuum silicidation. To address the long-standing problem of preparing a good electrical contact with Mg_2_Si, a CaSi_2_ layer with additionally provided high optical transparency was used as the top electrode. The resultant prototype solar cell with the b-SS/Fe_3_O_4_/Si/Mg_2_Si/CaSi_2_ structure demonstrated photoelectric conversion efficiency of 7.5% under AM1.5 illumination, which sets a new milestone for Si/Mg_2_Si based photovoltaics devices, approaching the 10% record ever reported for Si-silicide heterojunction solar cells. The described concept offers a feasible solution for building-integrated photovoltaics via utilization of earth abundant and construction compatible materials empowered by light-trapping structures. Further optimization of the developed SC and its performance could be achieved through careful optical modeling (for instance, by means of finite element calculations) that takes into account both the multi-scale layered morphology of the b-SS/Fe_3_O_4_/Si/Mg_2_Si/CaSi_2_ structure and the complex dielectric constants of its CaSi_2_ layer, which was not evaluated in the present study [53,54].

## Data Availability

Data will be made available on request.
